# Island-Type Graphene-Nanotube Hybrid Structures for Flexible and Stretchable Electronics: In Silico Study

**DOI:** 10.3390/mi14030671

**Published:** 2023-03-17

**Authors:** Michael M. Slepchenkov, Pavel V. Barkov, Olga E. Glukhova

**Affiliations:** Institute of Physics, Saratov State University, 410012 Saratov, Russia

**Keywords:** graphene-carbon nanotube hybrid films, island topology, density functional tight-binding method, axial stretching/compression, electron charge density, Landauer-Buttiker formalism, current-voltage characteristics, stretchable electronics

## Abstract

Using the self-consistent charge density functional tight-binding (SCC-DFTB) method, we study the behavior of graphene-carbon nanotube hybrid films with island topology under axial deformation. Hybrid films are formed by AB-stacked bilayer graphene and horizontally aligned chiral single-walled carbon nanotubes (SWCNTs) with chirality indices (12,6) and 1.2 nm in diameter. In hybrid films, bilayer graphene is located above the nanotube, forming the so-called “islands” of increased carbon density, which correspond to known experimental data on the synthesis of graphene-nanotube composites. Two types of axial deformation are considered: stretching and compression. It has been established that bilayer graphene-SWCNT (12,6) hybrid films are characterized by elastic deformation both in the case of axial stretching and axial compression. At the same time, the resistance of the atomic network of bilayer graphene-SWCNT (12,6) hybrid films to failure is higher in the case of axial compression. Within the framework of the Landauer-Buttiker formalism, the current-voltage characteristics of bilayer graphene-SWCNT (12,6) hybrid films are calculated. It is shown that the slope of the current-voltage characteristic and the maximum values of the current are sensitive to the topological features of the bilayer graphene in the composition of graphene-SWCNT (12,6) hybrid film. Based on the obtained results, the prospects for the use of island-type graphene-nanotube films in flexible and stretchable electronic devices are predicted.

## 1. Introduction

Over the past decades, carbon nanotubes (CNTs) and graphene have been the most discussed members of the nanocarbon allotropes family [1,2]. The unique mechanical, electronic, and thermal properties of these carbon nanomaterials open up wide opportunities for their applications in nano- and optoelectronics, electrochemistry, sensorics, and power engineering [3,4]. At the same time, several difficulties arise when using graphene and CNTs in various electronic and technical applications. For example, (1) the tendency of graphene sheets to aggregate during the synthesis of multilayer graphene by the reduction of graphene oxide [5]; (2) the presence of a large number of surface defects in the form of grain boundaries in the polycrystalline structure of monolayer graphene obtained by chemical vapor deposition (CVD) method [6]; (3) rather large values of contact resistance between CNTs and other conductors in carbon-based electronic devices [7]. One of the solutions to the problems mentioned above is the combination of graphene and CNTs in a single hybrid structure.

Using technologies such as CVD, solution processing, layer-by-layer self-assembly, and vacuum filtration, graphene-CNT hybrid structures with various topological configurations are synthesized [8,9,10,11]. Depending on the topological features, there are several classes of graphene-CNT hybrid structures. For example, CNT-rich and graphene-rich hybrid structures are distinguished [11]. CNT-rich hybrid structures can serve as excellent oxygen-reduction electrocatalysts [12]. Among graphene-rich hybrid structures, two categories are distinguished: hybrids with horizontally aligned CNTs, and hybrids with vertically aligned CNTs [11]. Hybrid structures with horizontally aligned CNTs are a promising material for flexible electronics, all-carbon transistors, optoelectronics, photovoltaics, and field emitter [13,14,15,16,17,18,19,20], while hybrid structures with vertically aligned CNTs are promising for hydrogen storage, supercapacitor electrodes, and thermal interface materials [21,22,23,24,25,26,27]. Hybrid structures with horizontally aligned CNTs are the most common type of graphene-CNT hybrids [8]. Differences in the chirality of CNTs and the type of bond between graphene and CNTs in the hybrid suggest a variety of possible topological configurations of the graphene-CNT hybrid structures. In this regard, research using computer modeling methods acquires special value. They make it possible to predict which configuration of the graphene-CNT hybrid structure is characterized by more advantageous properties.

Quite a lot of papers are devoted to the study of mechanical, thermal, and electronic properties of graphene-CNT hybrid structures with vertically aligned CNTs using computational modeling methods [28,29,30,31,32,33,34,35]. Of great interest to researchers is the theoretical study of the electronic and transport properties of graphene-CNT hybrids with horizontally aligned CNTs [36,37,38,39,40,41,42,43,44]. In these works, authors considered graphene-CNT hybrid structures both with a covalent bond between graphene and CNT, and with the interaction between them through van der Waals forces. The electronic properties of the graphene-nanotube hybrid structure have been studied during rotation and rigid displacement of the nanotube with respect to the underlying graphene layer [36]. It is shown that even at a vanishing twist angle, rigid displacements of a nanotube relative to a graphene substrate can qualitatively change its electronic structure. Studies of the electronic properties of graphene-CNT (12,0) hybrid structures with covalent attachment of a graphene sheet to a horizontally aligned CNT were carried out in several papers [38,39,40]. The calculation results showed that structures of this type are characterized by the appearance of Van Hove singularities in the distribution of the density of states, as well as an energy gap on the order of several hundred meV. The study of electron transport phenomena for van der Waals bonded graphene-CNT contacts based on (8,0) and (10,0) nanotubes was carried out by Cook et al. [41]. It has been established that the charge redistribution between graphene and CNTs leads to a rather low height of the Schottky barrier between CNTs and graphene, namely 0.09 eV and 0.04 eV for CNTs (8,0) and (10,0) respectively. It has been shown that the size of the overlap region between graphene and CNTs, as well as the diameter of the nanotube, have a significant effect on the size of the transport gap. Srivastava and Gaur analyzed changes in the electronic structure and transport properties of graphene-SWCNT hybrids with semiconductor and metal nanotubes as the distance between the SWCNT and graphene decreases. It was found that hybrids with semiconductor (13,0), (16,0) and metal (8,8), (12,0) nanotubes demonstrate different behavior at low source-drain bias voltages (up to 0.5 V) and similar behavior at higher voltages [42].

At the same time, in the above-mentioned articles, only non-chiral SWCNTs were used in the construction of topological models of graphene-CNT hybrid structures, while the majority of SWCNTs obtained during real synthesis are chiral nanotubes with a diameter of 0.7–1.2 nm. In addition, in a real experiment on the synthesis of graphene-CNT hybrids, structures with areas of increased density of carbon atoms are often obtained. This article discusses atomic models of graphene-nanotube hybrid structures with chiral SWCNTs (12,6) of subnanometer diameter. We carry out predictive studies of the behavior of such structures under the stretching/compression deformations and evaluate how the electrically conductive properties change in this case.

## 2. Calculation Details

To calculate the atomic structure and energy parameters of graphene-SWCNT (12,6) hybrid films, we used the self-consistent charge density functional tight-binding (SCC-DFTB) method [45] implemented in the atomistic quantum mechanical software DFTB+ version 20.2 [46] (University of Bremen, Bremen, Germany). At the stage of calculating the total energy of the system, the tight binding approximation is used. It is included in the density functional theory (DFT) model using perturbation theory. The SCC-DFTB method considers the influence of electron density fluctuations on the total energy of the system. The distribution of the electron charge density over atoms is determined using the Mulliken population analysis scheme [47]. Considering the self-consistent charge distribution makes it possible to substantially improve the accuracy of calculations for polyatomic systems. The SCC-DFTB model uses the valence approximation. According to this approximation, the largest contribution to the total energy is made by valence orbitals. They are described in terms of the basis of Slater-type orbitals with the set of parameters pbc-0-3 [46]. The choice of the SCC-DFTB method for calculating the total energy of a system is due to the polyatomic nature of the supercells of graphene-SWCNT (12,6) hybrid films.

The electrical conductivity *G* was calculated within the framework of the Landauer-Buttiker formalism [48] according to the formula

(1) $$G=\frac{I}{V}=\frac{2e^{2}}{h}\int_{-\infty }^{\infty }T\left(E\right)F_{T}\left(E-E_{F}\right)dE,$$

where *T*(*E*) is the electron transmission function, *F_T_* is the function that determines the temperature broadening of the energy levels, *E_F_* is the Fermi energy of the contacts, *e* is the electron charge, *h* is Planck’s constant, 2*e*^2^/*h* is the value of the conductance quantum doubled to consider the spin. The electron transmission function *T*(*E*) is given by:

(2) $$T\left(E\right)=\frac{1}{N}\sum_{k=1}^{N}\mathrm{Tr}(\Gamma_{S}\left(E\right)G_{C}^{A}\left(E\right)\Gamma_{D}\left(E\right)G_{C}^{R}\left(E\right)),$$

where $G_{C}^{A}\left(E\right)$, $G_{C}^{R}\left(E\right)\;$ are the leading and retarded Green’s matrices describing the contact with the electrodes, $\Gamma_{S}\left(E\right)$, $\Gamma_{D}\left(E\right)$ are the level-broadening matrices for the source and drain.

Since the supercells of the studied graphene-nanotube structures contain 250–300 atoms, an original method was used to accelerate the calculation of the transmission function *T(E)* [49]. This method makes it possible to calculate the function *T(E)* for a small number of *k*-points of the first Brillouin zone, and then interpolate it for any *k*-point of the first Brillouin zone and restore the full transmission function *T*(*E*). All calculations were carried out at a temperature of 300 K.

## 3. Results

### 3.1. Topological Models of Bilayer Graphene-SWCNT (12,6) Hybrid Structures of Island Type

**Figure 1:**
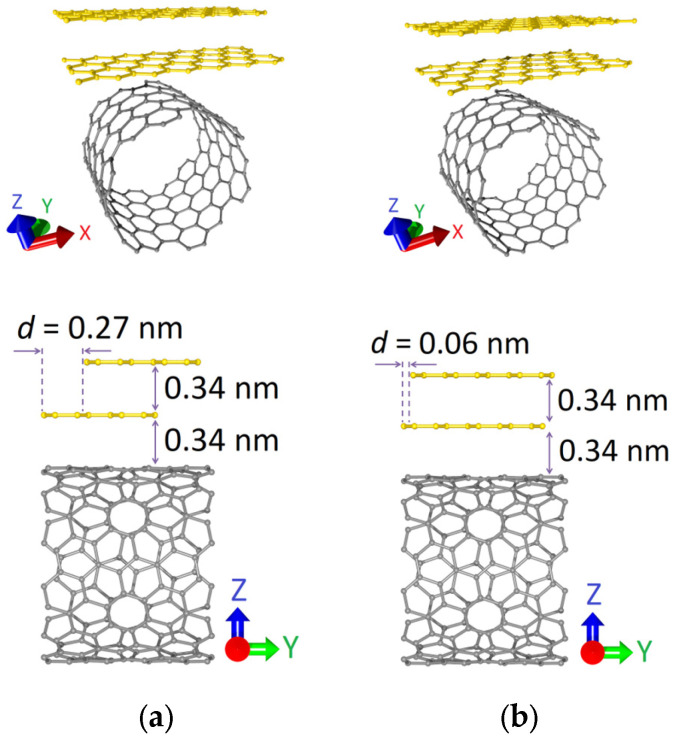
Atomic structure of the initial (before optimization) configurations of the supercells of bi-layer graphene-SWCNT (12,6) hybrid films with island topology: (**a**) model V1; (**b**) model V2.

Within the framework of this study, two topological models of graphene-SWCNT hybrid films were considered. The supercell of each model was a 2D structure formed by an AB-stacked bilayer zigzag graphene nanoribbon (ZGNR) and SWCNT with chirality indices (12,6). The bilayer graphene was located above the surface of the carbon nanotube perpendicular to its axis, forming the so-called “islands” of increased carbon density in the composition of the hybrid structure. Figure 1 shows the atomic structure of the initial (before optimization) configurations of the supercells of bilayer graphene-SWCNT (12,6) hybrid films. The difference between the models was in the ZGNR width: in the first case (model V1) it was 0.71 nm (3 hexagons), and in the second case (model V2) it was 0.92 nm (4 hexagons). In proportion to the width of the nanoribbon, the shift between graphene layers *d* along the Y axis (along the nanotube axis) also changed: *d* = 0.27 nm for model V1 and *d* = 0.06 nm for model V2. The distance between graphene layers along the Z axis (perpendicular to the nanotube axis) was the same for both topological models and amounted to 0.34 nm.

**Figure 2:**
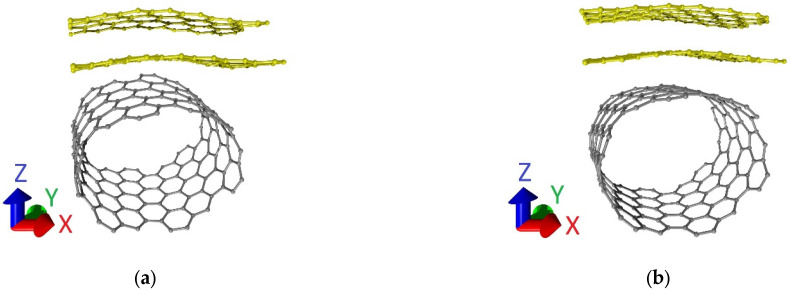
Supercells of bilayer graphene-SWCNT (12,6) hybrid films with island topology: (**a**) model V1; (**b**) model V2.

The supercells of bilayer graphene-SWCNT (12,6) hybrid films with an island topology after optimization of the atomic structure (finding of the equilibrium configuration) are shown in Figure 2. Comparing Figure 1 and Figure 2, it can be noted that both the nanotube (12,6) and the bilayer graphene as part of the supercells of models V1 and V2 are deformed during the optimization of the atomic structure of the supercell. Translation vectors of supercells of the considered topological models: *L_x_* = 1.707 nm and *L_y_* = 1.110 nm for model V1, *L_x_* = 1.723 nm, and *L_y_* = 1.134 nm for model V2. Previously, based on the results of calculating the binding energy, it was found that the supercells of the considered topological models are energetically stable [50].

The main objective of our study was to reveal the regularities in the behavior of bilayer graphene-SWCNT (12,6) hybrid films with island topology under mechanical deformations and to evaluate how their electrically conductive properties would change in this case. We considered two types of deformation: axial stretching and axial compression. Their choice is due to the fact that in the design of flexible and stretchable electronic devices, functional nanomaterials are tested precisely for these types of deformation.

### 3.2. Axial Stretching Deformation

**Figure 3:**
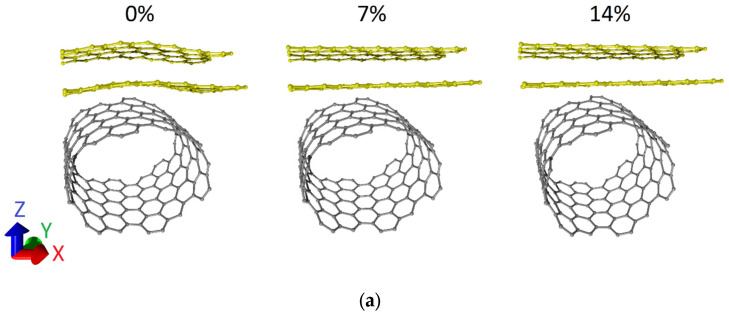

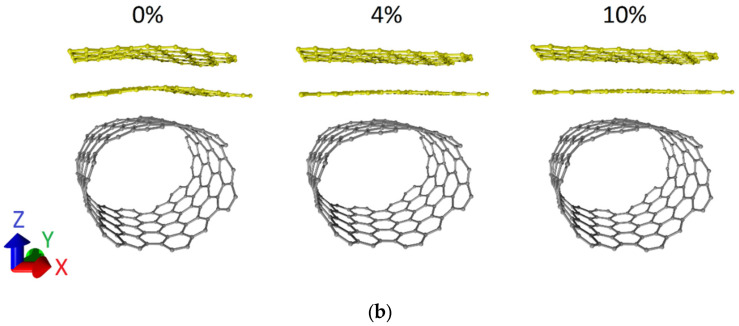
Atomic configurations of supercells of bilayer graphene-SWCNT (12,6) hybrid films with island topology at various values of axial stretching deformation: (**a**) model V1; (**b**) model V2.

Let us turn to the consideration of the case of axial stretching deformation. In the course of the numerical experiments, we simulated the stretching of supercells of bilayer graphene-SWCNT (12,6) hybrid films along the X-axis, i.e., along the zigzag edge of bilayer graphene. This variant of deformation application is due to the following reason. The supercell of the 2D hybrid structure of bilayer graphene-SWCNT (12,6) includes a fragment of bilayer graphene with different side lengths in the X and Y directions. The difference between the graphene sizes along the X and Y axes is about 3 times. Therefore, we can say that the bilayer graphene in a supercell has the shape of a nanoribbon with an extended side along the X-axis. In this regard, it is logical to assume that the stresses of the atomic network will differ greatly when a load was applied to the graphene side with a smaller extension (along the Y axis) and when a load was applied to the graphene side with a larger extension (along the X axis). Step-by-step deformation was carried out. At each step, the length of the translation vector *L_x_* of supercells was increased by 1%. Then, the search for the equilibrium configuration of the deformed structure was carried out by optimizing the geometric parameters of the supercell. Based on the results of the numerical simulations, the stretching strength limits of the topological models were determined. Figure 3 shows the atomic configurations of supercells of bilayer graphene-SWCNT (12,6) hybrid films at different deformations. For both topological models, one can note a tendency for the atomic network of graphene layers to straighten as it is stretched. Model V1 is more resistant to failure when stretched along the X-axis. For it, the breaking of covalent bonds between the atoms of the ZGNR occurred upon stretching by 15%. For the V2 model, the bond breaking occurred upon stretching by 11%. To establish the nature of deformation during the stretching of supercells of bilayer graphene-SWCNT (12,6) hybrid films, we calculated the dependences of the strain energy *E_str_* on the stretching value ΔL in relative units. The strain energy *E_str_* was calculated according to the formula:

(3) $$E_{\mathrm{str}}=\left(E_{\mathrm{tot}1}-E_{\mathrm{tot}2}\right)/N,$$

where *E_tot_*_1_ is the total energy of the supercell before stretching, *E_tot_*_2_ is the total energy of the supercell after stretching, and *N* is the number of atoms in the supercell.

**Figure 4:**
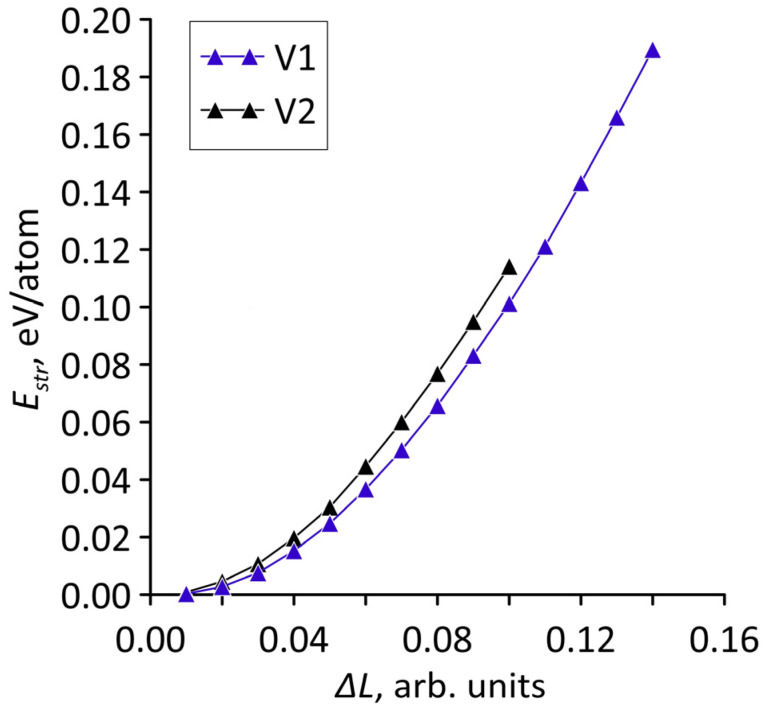
Dependence of the strain energy of bilayer graphene-SWCNT (12,6) hybrid films on the magnitude of stretching in relative units for supercells of topological models V1 and V2.

The calculated dependencies are shown in Figure 4. Since the number of atoms in the supercells of the considered topological models is different, the strain energies are given in eV/atom. It can be seen that for both models there is a quadratic increase in the strain energy with stretching. Consequently, over the entire considered range of deformations, elastic deformation is typical for models V1 and V2.

**Figure 5:**
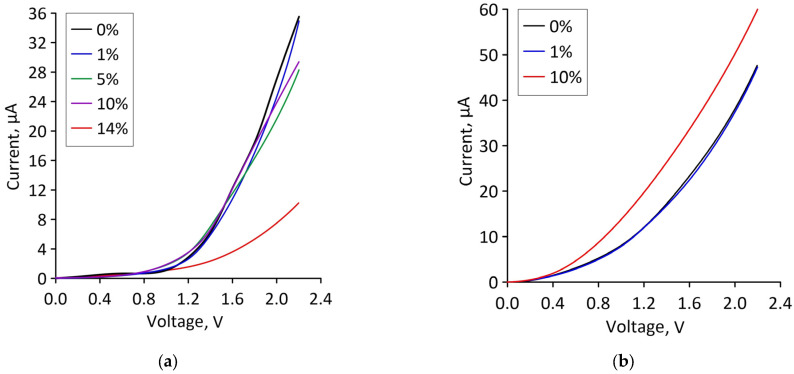
Current-voltage characteristics of bilayer graphene-SWCNT (12,6) hybrid films with island topology for various cases of axial stretching: (**a**) model V1; (**b**) model V2.

Let us turn to reveal the patterns of current flow in bilayer graphene-SWCNT (12,6) hybrid films with island topology under deformation. Since axial stretching along the X axis (along the zigzag edge of the graphene bilayer) was considered in the framework of the numerical experiments, electron transport calculations were also carried out only for the case of current transfer along the X axis. For the operation of stretchable electronic devices, the current characteristics of materials are of interest. Figure 5 shows current-voltage (*I–V*) characteristics of bilayer graphene-SWCNT (12,6) hybrid films for topological models V1 and V2 under stretching by different percentages. For model V1, the *I–V* characteristic practically does not change in the strain range of 1–10%. A decrease in the slope of the *I–V* characteristic and the maximum current values is observed only at 14% stretching, i.e., immediately before breaking the covalent bonds between atoms in the graphene bilayer. For model V2, the slope of the *I–V* characteristic increases with increasing stretching, and the maximum current value is almost two times higher than the maximum current value of model V1.

**Figure 6:**
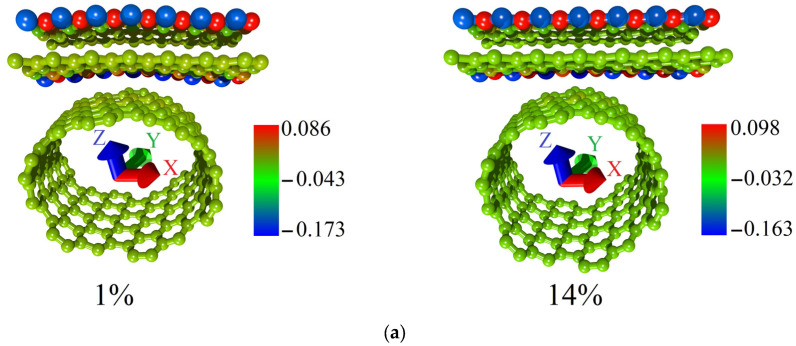

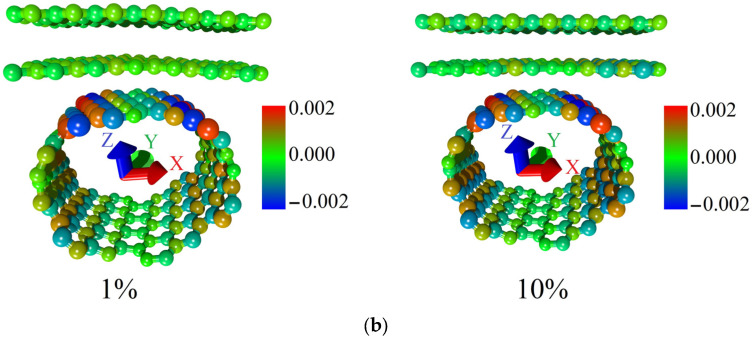
Distribution of the electron charge density over the supercell atoms of bilayer graphene-SWCNT (12,6) hybrid films with island topology for the cases of minimum and maximum allowable axial stretching: (**a**) model V1; (**b**) model V2.

To explain this difference in the behavior of the topological models V1 and V2, we performed a Mulliken Population Analysis. The calculated distributions of the electron charge density over the atoms of supercells of models V1 and V2 for the cases of the minimum and maximum allowable strain are shown in Figure 6. The fraction of the electron charge is given in units of |e| in the figure. Comparing the distributions for models V1 and V2, it can be noted that in the case of model V1 (Figure 6a), a significant fraction of the electron charge from the graphene bilayer atoms was transferred to the nanotube atoms. In the case of model V2 (Figure 6b), the charge on the graphene bilayer is not transferred to the nanotube. Figure 6b demonstrates that the redistribution of the electron charge density during axial stretching occurs only between nanotube atoms. Since the current-voltage characteristic calculations were carried out for the case of electron transport along the graphene bilayer (along the X-axis), the charge conservation on graphene is a key factor in explaining the highest current value for model V2.

Thus, model V1 demonstrates a higher resistance to failure when stretched along the X-axis while maintaining electrically conductive properties.

### 3.3. Axial Compression Deformation

**Figure 7:**
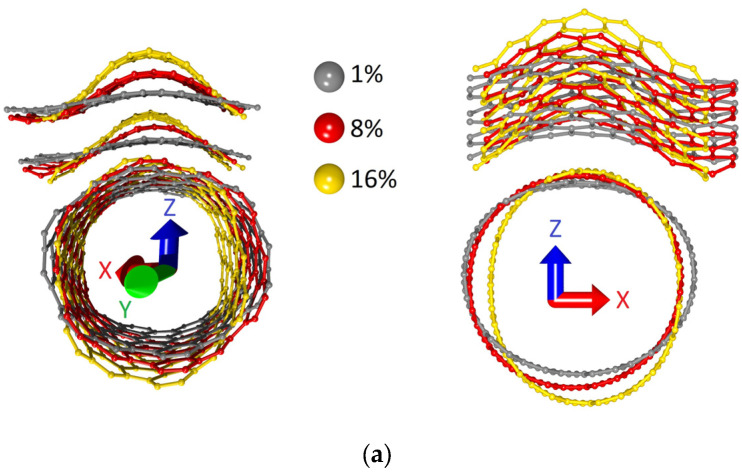

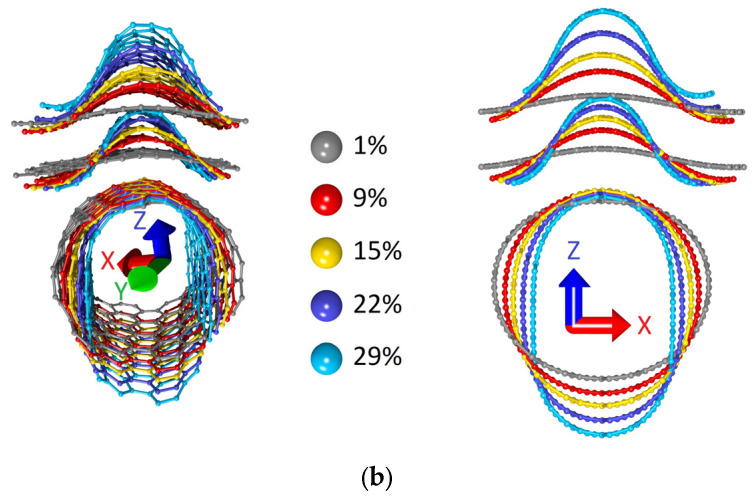
Changes in the atomic network of supercells of bilayer graphene-SWCNT (12,6) hybrid films under axial compression: (**a**) model V1; (**b**) model V2.

**Figure 8:**
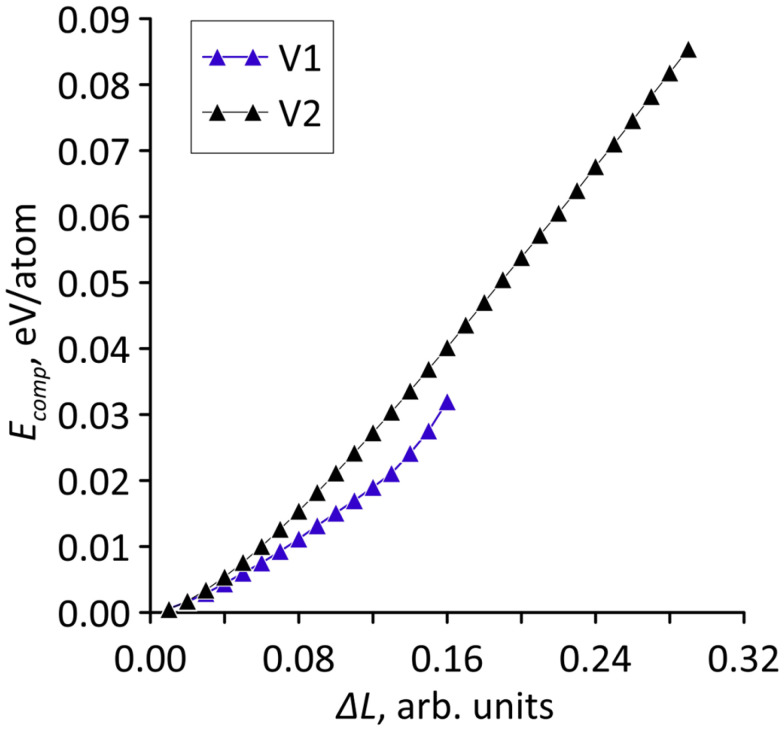
Dependence of the strain energy of bilayer graphene-SWCNT (12,6) hybrid films on the magnitude of compression in relative units for supercells of topological models V1 and V2.

The study of the effect of axial compression on the structural and electrically conductive properties of bilayer graphene-SWCNT (12,6) hybrid films was carried out according to the same plan as the study of the effect of axial stretching. Stepwise compression of supercells of models V1 and V2 along the zigzag edge of the graphene bilayer (along the X-axis) was simulated. At each deformation step, the supercell length along the X-axis (*L_x_*) decreased by 1%. The pattern of changes in the atomic network of supercells at different values of axial compression is shown in Figure 7. The calculated dependences of the strain energy *E_comp_* on the axial compression in relative units for the models V1 and V2 are shown in Figure 8. Figure 7 shows that the supercells of both models behave similarly under axial compression: the bilayer graphene takes on a wave-like shape with an amplitude that increases with compression, and the nanotube takes on the shape of an elongated ellipsoid. At the same time, the high resistance to failure on compression of model V2 attracts attention. For it, the moment of destruction of covalent bonds between atoms occurs only upon compression by 29% of its initial dimension, while for model V1, bond breaking occurs already upon compression by 16% of its initial dimension. The dependences in Figure 8 demonstrate a quadratic increase in the strain energy with increasing compression, which indicates elastic deformation for the bilayer graphene-SWCNT (12,6) hybrid films.

**Figure 9:**
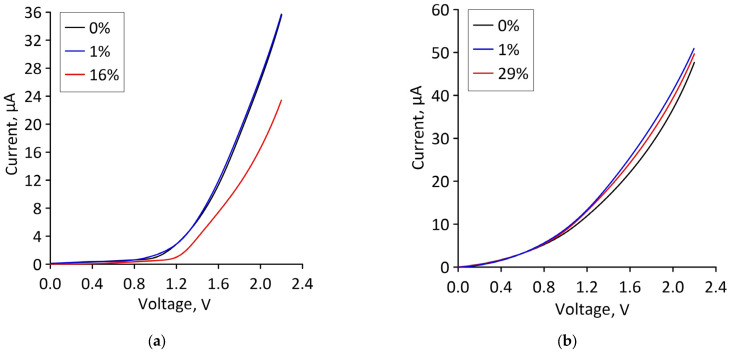
Current-voltage characteristics of bilayer graphene-SWCNT (12,6) hybrid films with island topology for various cases of axial compression: (**a**) model V1; (**b**) model V2.

Let us now find out how a change in the atomic structure of a supercell under axial compression affects the ability of bilayer graphene-SWCNT (12,6) hybrid films to conduct electric current. The calculated current-voltage characteristics of supercells of models V1 and V2 for the cases of minimum and maximum axial compression, as well as in the absence of deformation, are shown in Figure 9. It can be seen that for model V1, in the case of ultimate strain (compression by 16%), the slope of the current-voltage characteristics and the maximum current value decrease quite noticeably compared to their values at the initial strain. For model V2, the slope of the current-voltage characteristic and the maximum value of the current almost do not change when moving from the minimum deformation to the limit.

**Figure 10:**
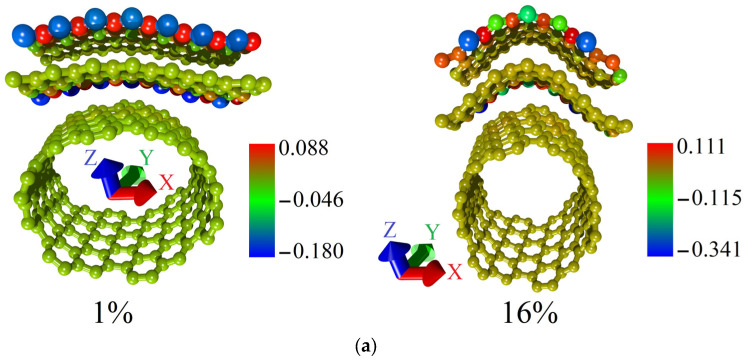

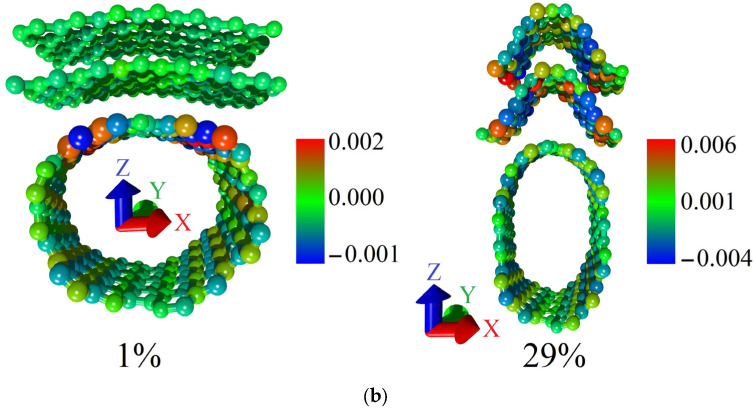
Distribution of the electron charge density over the supercell atoms of bilayer graphene-SWCNT (12,6) hybrid films with island topology for the cases of minimum and maximum allowable axial compression: (**a**) model V1; (**b**) model V2.

Comparing the maximum current values at a voltage of 2.2 V for supercells of models V1 and V2, it can be seen that for model V2, the maximum current is almost two times higher than for model V1. The same trend was found for the V2 model in the case of axial stretching. To clarify this result, we calculated the distributions of the electron charge density over the atoms of the supercells of models V1 and V2 in the cases of minimal and ultimate axial compression. They are shown in Figure 10. The presented distributions clearly show that in the case of the V1 model, the charge from the atoms of the graphene bilayer partially transfers to the nanotube, while for the V2 model, there is no charge transfer from graphene to the nanotube. Since the calculation of the current-voltage characteristics was carried out for the case of electron transport along the graphene bilayer, the larger value of the electron charge density on graphene atoms determines the larger current in this direction.

Thus, the results of simulating axial compression showed that the model V2 of bilayer graphene-SWCNT (12,6) hybrid films is characterized by the highest resistance to failure on compression, as well as maximum current values and negligibly small changes in the current-voltage characteristics with increasing strain.

## 4. Discussion

**Figure 11:**
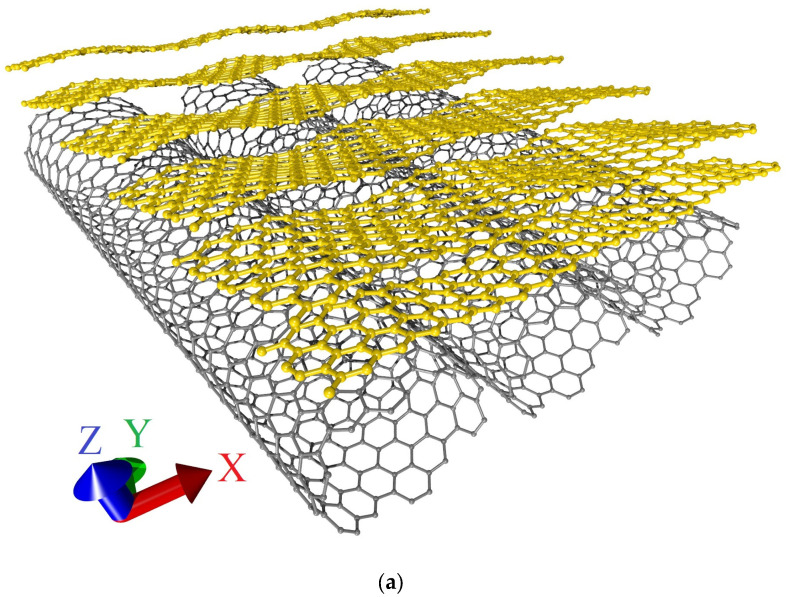

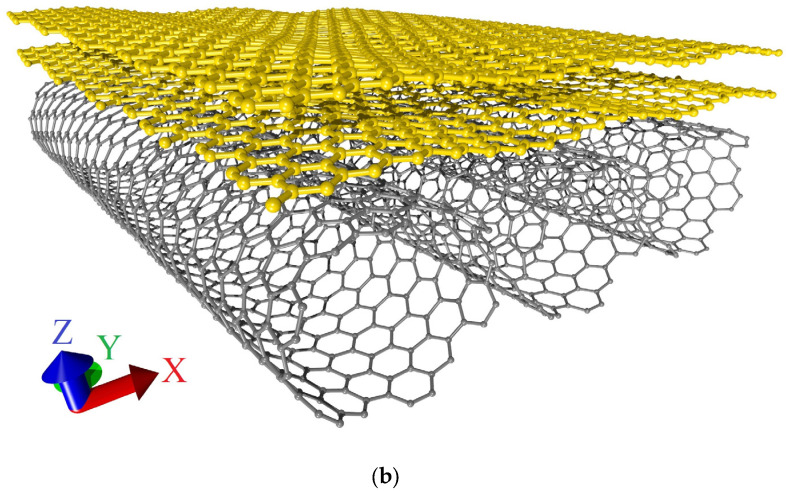
Extended fragments of the atomic structure of bilayer graphene-SWCNT (12,6) hybrid films with island topology: (**a**) model V1; (**b**) model V2.

Discussing what could cause such differences between the behavior of models V1 and V2 both under axial compression and under axial stretching, we analyzed the topology of the extended fragments of the atomic structure of each model obtained by translating their supercells (Figure 11).

Figure 11 shows that in the case of model V1, the graphene layers in the form of nanoribbons line up one after another at an angle with respect to the nanotube surface in an extended structural fragment. The edge atoms of one layer are located above the edge atoms of the other layer, inducing a redistribution of the electron charge density between them and the nanotube, as well as a curvature of their atomic network. In the extended fragment of model V2, the graphene layers no longer take the form of nanoribbons, but the form of sheets with similar dimensions in both directions. In this case, the atomic network of graphene layers has a minimum curvature, and the interaction of layers at the level of electron orbitals is much weaker as a result. It can be assumed that the transition from graphene nanoribbons to graphene sheets as part of a bilayer graphene-SWCNT (12,6) hybrid structure with an island topology is the reason for such noticeable differences in the response of topological models V1 and V2 to axial stretching and compression.

**Figure 12:**
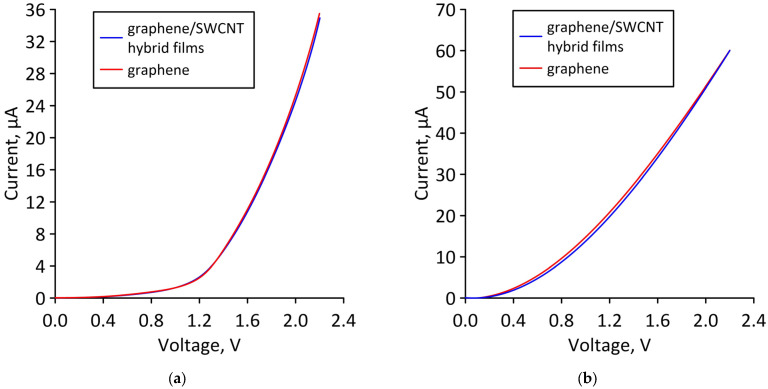
Current-voltage characteristics of bilayer graphene-SWCNT (12,6) hybrid films with island topology: (**a**) model V1 at axial stretching by 14%; (**b**) Model V2 at axial stretching by 10%.

**Figure 13:**
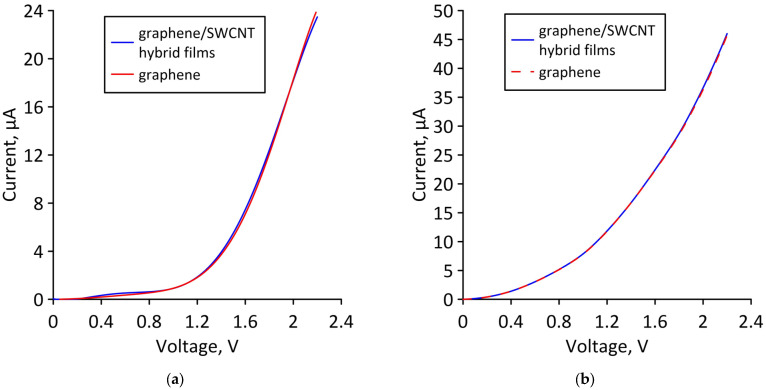
Current-voltage characteristics of bilayer graphene-SWCNT (12,6) hybrid films with island topology: (**a**) model V1 at axial compression by 16%; (**b**) model V2 at axial compression by 29%.

To confirm our assumption, let us compare the current-voltage characteristics of a bilayer graphene-SWCNT (12,6) hybrid film and AB-staked bilayer graphene calculated for the limiting cases of axial stretching/compression. Figure 12 shows the current-voltage characteristics of a hybrid film and AB-staked bilayer graphene of models V1 and V2 for the case of axial stretching, in which the covalent bonds between carbon atoms in the supercell are broken (stretching by 14% for model V1 and by 10% for model V2). Figure 13 shows the current-voltage characteristics of a hybrid film and AB-staked bilayer graphene of models V1 and V2 for the case of axial compression, in which the covalent bonds between carbon atoms in the supercell are broken (compression by 16% for model V1 and by 29% for model V2). Figure 12 and Figure 13 clearly demonstrate that both in the case of axial stretching and in the case of axial compression, the current-voltage characteristics of the AB-staked bilayer graphene and the bilayer graphene-SWCNT (12,6) hybrid film completely coincide. Consequently, the almost twofold difference in current values between models V1 (with a ZGNR width of 3 hexagons) and V2 (with a ZGNR width of 4 hexagons) is explained precisely by the difference in the AB-staked bilayer graphene topology in them, since the degree of deformation of the nanotube (12,6) in both models are the same.

Hence, the key topological parameters from the standpoint of controlling the electrically conductive properties of bilayer graphene-SWCNT films (12,6) hybrid structures are the width of the bilayer graphene nanoribbon and the magnitude of the shift of the graphene layers in the supercell.

## 5. Conclusions

Thus, we have studied the deformation behavior and electrically conductive properties of bilayer graphene-SWCNT (12,6) hybrid films with an island topology under axial stretching and compression. It has been found that the topology of a graphene bilayer in a supercell is a key factor in determining the response of a bilayer graphene-SWCNT (12,6) hybrid structure to axial stretching/compression. The topological model of a bilayer graphene-SWCNT (12,6) hybrid film with a ZGNR width of 3 hexagons as part of a supercell is characterized by the highest resistance to failure on axial stretching and stability of electrically conductive properties. The topological model of a bilayer graphene-SWCNT (12,6) hybrid film with a bilayer nanoribbon width of 4 hexagons as part of a supercell has the highest resistance to failure on axial compression, a stable current-voltage characteristic slope, and maximum current values. By controlling the topology of bilayer graphene in the composition of island-type hybrid graphene-nanotube structures, it is possible to achieve optimal values of their electrically conductive characteristics for subsequent use in flexible and stretchable electronic devices, in particular, as conductive electrodes.

## Data Availability

Not applicable.
